# Visual Impairment Prevalence, Causes, and Role of Healthcare Access: A Systematic Review and Meta-Analysis in Iran

**DOI:** 10.1155/2020/4710328

**Published:** 2020-11-23

**Authors:** Mehrdad Afarid, Hossein Molavi Vardanjani, Hamideh Mahdaviazad, Marzieh Alamolhoda, Saman Farahangiz

**Affiliations:** ^1^Poostchi Ophthalmology Research Center, Department of Ophthalmology, Medical School, Shiraz University of Medical Sciences, Shiraz, Iran; ^2^School of Medicine, Shiraz University of Medical Sciences, Shiraz, Iran; ^3^Community and Preventive Medicine, Poostchi Ophthalmology Research Center, Medical School, Shiraz University of Medical Sciences, Shiraz, Iran; ^4^Community Medicine, MPH Department, Shiraz University of Medical Sciences, Shiraz, Iran

## Abstract

**Purpose:**

Healthcare access is one of the determinants of visual impairment (VI), as a public health problem. The objective of this study was to estimate VI prevalence, related causes, and its correlation with access to physicians in Iran.

**Methods:**

: This systematic review and meta-analysis include observational studies published in Iran. PubMed, Web of Science, Scopus, Google Scholar, and local databases were systematically searched by using the MeSH headings. Data on the provincial distribution of physicians, as an index of access to healthcare, was retrieved. A random-effect meta-analysis was performed to assess.

**Results:**

Eight articles were included. The pooled prevalence of blindness, low vision, and VI was 0.80% (95% CI: 0.61–0.99%), 2.92% (95% CI: 2.40–3.44%), and 5.57% (95% CI: 4.71–6.43%). Refractive errors were the most common causes of VI based on PVA with the pooled prevalence of 54.6% (95% CI: 43.4–65.8%). Based on BCVA, we found that the pooled prevalence of cataracts was 37.4% (95% CI: 29.5–45.3%) as the most common cause of VI. The results of metaregression showed that the greater number of general practitioners (GPs) (*P* value = 0.01) and pharmacists (*P* value = 0.024) per population were associated with a lower prevalence of blindness.

**Conclusion:**

Some of the main causes of visual impairment in Iran are preventable. Access to healthcare services may lead to early diagnosis of preventable causes of VI. Further well-designed studies and national surveys should be conducted to provide accurate data from different regions of Iran.

## 1. Introduction

Visual impairment (VI) is one of the most common public health issues with substantial personal, financial, and social burdens on both patients and healthcare systems [1–3]. It is estimated that there are 285 million people with VI globally, of which 39 million are blind and the remaining are affected by low vision [4, 5].

Generally, cases of low vision and blindness comprise VI as the main category. The definition of low vision is “visual acuity of less than 6/18 but equal to or better than 3/60, or a corresponding visual field loss to less than 20°, in the better eye with the best possible correction” and blindness is defined as “visual acuity of less than 3/60, or a corresponding visual field loss to less than 10°, in the better eye with the best possible correction. [6].

The main related causes of VI are refractive errors, cataract, macular degeneration, and glaucoma [3, 7, 8]. It has been shown that the causes of VI vary in developed and developing countries. Most of the known causes of VI are preventable and manageable by medical treatments, interventions, or access to primary healthcare services. In general, primary healthcare as the main mechanism of the healthcare service delivery is the first level of relationship between the individual, family, and community with the health system of countries. Primary healthcare services are located in the community and refer patients to other levels of healthcare services. Physicians work in secondary and tertiary healthcare centers. In each geographic region, the number and distribution of physicians are one of the indices of access to healthcare services. Improving the quality of healthcare services and increasing access to health services for community members especially for people with lower socioeconomic status are the most important goals of healthcare in Iran [9].

In 1999, the World Health Organization (WHO) in partnership with the International Agency for the Prevention of Blindness (IAPB) initiated “VISION 2020: the Right to Sight” with the aim to abolish avoidable blindness [10–12]. Eye care services at a higher level are usually carried out in hospitals or dispensaries at the district or provincial level. This level is integrated into the general medical infrastructure that uses all of the sophisticated equipment and existing staff such as ophthalmic assistants, general practitioners (GPs) trained in eye care, or fully qualified ophthalmologists [13]. However, the lack of physicians and the unequal geographical distribution of the workforce are major barriers to the population's access to eye care services in Iran [14].

In recent years, a number of epidemiologic studies have been developed to report VI prevalence and its related causes. However, in most of the aforementioned studies, there is a significant heterogeneity of prevalence across the reports on VI prevalence and its related causes in various parts of Iran [1]. It can be attributed to the individual's uneven access to healthcare services within communities or a lack of access to necessary determinants of health [15]. Therefore, access to healthcare services could be considered as a potentially related factor to the prevalence of VI.

As most of the causes of VI and blindness are preventable and equitable access to primary healthcare is known to have a positive impact on preventing the risk factors and causes of diseases, we hypothesized that better access to healthcare services in a community including access to physicians is negatively related to VI prevalence in various regions of Iran. To the best of our knowledge, this is the first systematic synthesis of evidence and meta-analysis that assess the relationship between VI prevalence and causes to access to healthcare services. Therefore, in this study, we aimed to assess the VI prevalence, related causes, and its relation with access to physicians (as one of the main indices of healthcare access) in Iran.

## 2. Materials and Methods

This secondary study was conducted according to the Meta-Analyses and Systematic Reviews of Observational Studies (MOOSE) guideline during the process [16].

### 2.1. Literature Search Strategy

International and national databases including PubMed, Web of Science, Scopus, Google Scholar, SID (Scientific Information Database), and MagIran were systematically searched for observational studies. Based on the MeSH terms, appropriate keywords such as “epidemiology”, “prevalence”, “incidence”; terms related to the outcomes of interest such as “blindness”, “vision low”, “vision disorder”; and “Iran” were searched in the titles and abstracts. A manual search of the reference lists of review article and practice guidelines were executed to identify any additional studies.

### 2.2. Inclusion and Exclusion Criteria and Data Extraction

We selected articles with full text available based on the following criteria: (a) observational studies from the general population; (b) from all geographical regions of Iran; (c) in both Persian and English languages; (d) exploring the prevalence of VI, low vision, and blindness, as the study dependent variables; (e) investigating the dependent variables in an Iranian population from 1980 to 2017. Exclusion criteria were clinic-based studies, the same data being used in two separate studies, studies receiving less than six stars in the quality assessment process, and the studies with a sample that did not represent the general population. Two investigators (SF and HM) screened all retrieved studies independently in two phases including titles and abstracts and then full texts. Studies were categorized into three groups (relevant, irrelevant, and unclear). Disagreements were discussed with the third investigator and resolved. Finally, documents were relevant or irrelevant. Relevant documents were critically appraised. The Newcastle- Ottawa Quality Assessment Scale for observational studies and its staring system was used for quality and bias assessment. This assessment was conducted by two investigators independently and disagreements were discussed with a methodologist. The following data were extracted: (1) first author's last name; (2) study year; (3) location (rural/urban); (4) sets number; (5) sample size; (6) population age range; (7) response rate; (8) the outcome according to the WHO or US criteria; (9) VI assessment method (all VI assessment methods were based on the best-corrected visual acuity (BCVA), presenting visual acuity (PVA) and/or pinhole visual acuity); (10) study province; (11) sex ratio; (12) a number of registered GPs, pharmacists, medical specialists, and medical subspecialists working in the study province according to the most recent available reports by Iran's Ministry of Health and Medical Education (MOH) [14]. Data on the provincial distribution of physicians was retrieved as an index of access to healthcare (number 12). Access to physicians (GPs, pharmacists, medical specialists, and medical subspecialists) is a part of healthcare services. Based on the reports by MOH, the geographical distribution of physicians was considered as an index to access healthcare. Since there is a relationship between eye disorders and other diseases such as diabetes mellitus, hypertension, and cardiovascular pathologies, the study population consisted of the GPs, medical specialists, and medical subspecialists according to the nearest available reports by MOH to the time of selected studies for this analysis. This type of integrated approach is necessary to ensure a better knowledge of the comorbidities to prevent ocular disorders.

### 2.3. Statistical Analysis

Heterogeneity among studies was assessed using *Q*-statistic that is distributed as *χ*^2^ under the assumption of homogeneity of effect sizes and *I*^2^ index. *I*^2^ values ranged between 0 and 75%, which represented none to high heterogeneity. The overall pooled prevalence of blindness, low vision, and VI were estimated by using a random-effect model with DerSimonian and Laird method. A random-effect metaregression analysis was used to assess the associations between the geographic distribution of healthcare access and the overall pooled prevalence estimate. All statistical analyses were performed by Stata software version 12 (Stata Corporation, College Station, Texas, USA). *P* values less than 0.05 were considered to be statistically significant.

## 3. Results

**Figure 1:**
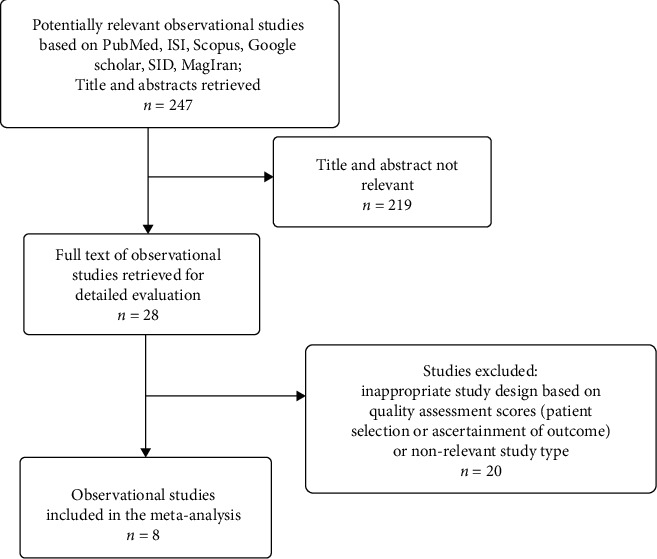
Flowchart of the process of study selection, which is based on MOOSE guideline.

Flowchart of study selection based on MOOSE guideline is shown in Figure 1. We initially screened 247 eligible abstracts. Of these, 219 were excluded due to duplication or irrelevancy during the title and abstract screening. Subsequently, 20 articles were excluded after reviewing the full text due to the irrelevancy of the study type or lack of quality (quality score < 6 stars). Finally, a total of eight studies were included in the present meta-analysis.

The characteristics of the eight included studies are summarized in Table 1. Publication years were 2004–2017, and the sample size varied from 1,185 to 11,975. Overall, five studies were conducted on all age groups and three studies on participants older than 40 years.

### 3.1. Prevalence of Blindness

**Figure 2:**
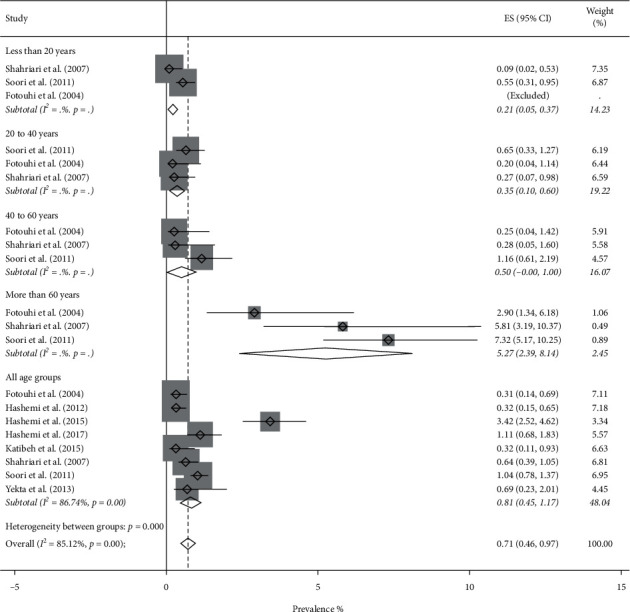
Forest plot of the prevalence of blindness among Iranian males.

**Figure 3:**
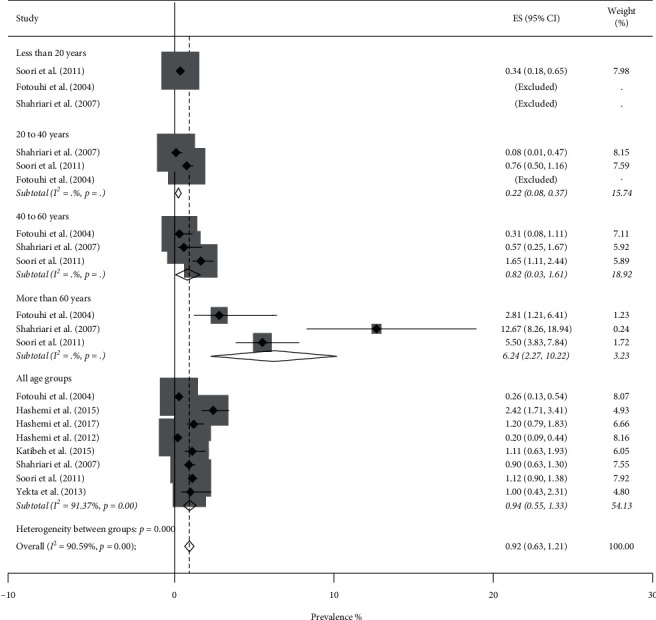
Forest plot of the prevalence of blindness among Iranian females.

Totally, the pooled prevalence of blindness using the random-effect model was 0.80% (95% CI: 0.61–0.99%, *Q* = 291.09, *I*^2^ = 87.98%). Figures 2 and 3 show the forest plot generated for men and women which is stratified by the age groups. Blindness prevalence in men was 0.71% (95% CI: 0.46–0.97%) and in women was 0.92% (95% CI: 0.63–1.21%).

Further, the results of metaregression revealed that a greater number of GPs (*t* = −3.45, *P* value = 0.001) and pharmacists (*t* = −2.30, *P* value = 0.024) was associated with a lower prevalence of blindness, while the greater number of medical specialists was associated with a higher prevalence of blindness (*t* = 3.58, *P* value = 0.001).

### 3.2. Prevalence of Low Vision

**Figure 4:**
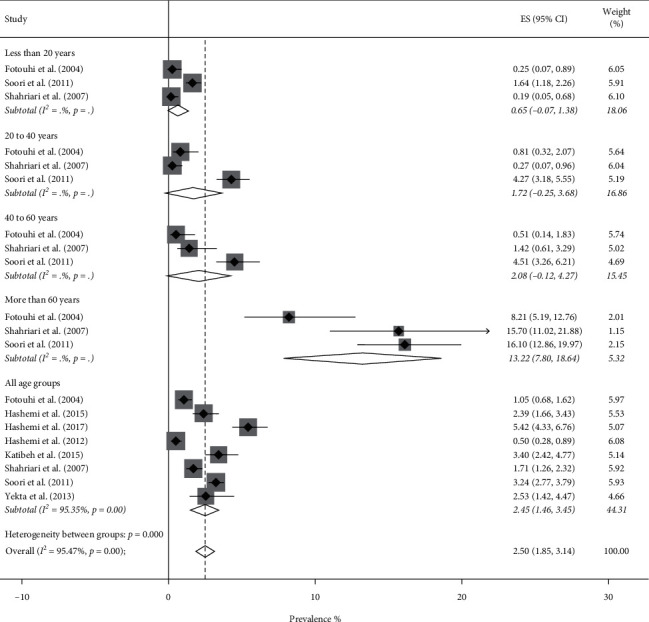
Forest plot of the prevalence of low vision among Iranian males.

**Figure 5:**
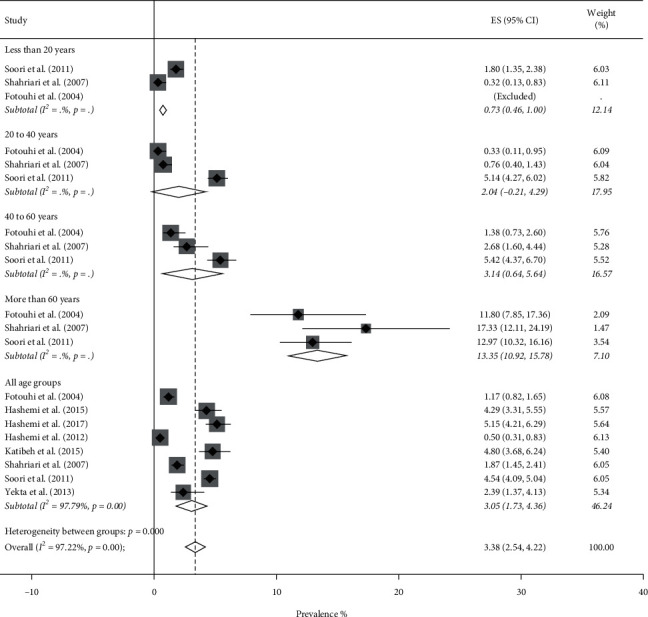
Forest plot of the prevalence of low vision among Iranian females.

The overall pooled prevalence of low vision was 2.92% (95% CI: 2.40–3.44%, *Q* = 1092.58, *I*^2^ = 96.52%). The results of the forest plots showed that Low vision prevalence rate was 2.50% (95% CI: 1.85–3.14%) in men and 3.38% (95% CI: 2.54–4.22%) in women. There was considerable variation in the prevalence of low vision across age groups in both genders. This indicates that the prevalence of low vision was significantly higher in the 60 years and older age group (Figure 4 for men and Figure 5 for women). The meta-regression model showed that the number of GPs was independently associated with a lower prevalence of low vision (*t* = −2.80, *P* value = 0.006), while a higher density of specialists was associated with a higher prevalence of low vision (*t* = 2.54, *P* value = 0.013).

### 3.3. Prevalence of VI

**Figure 6:**
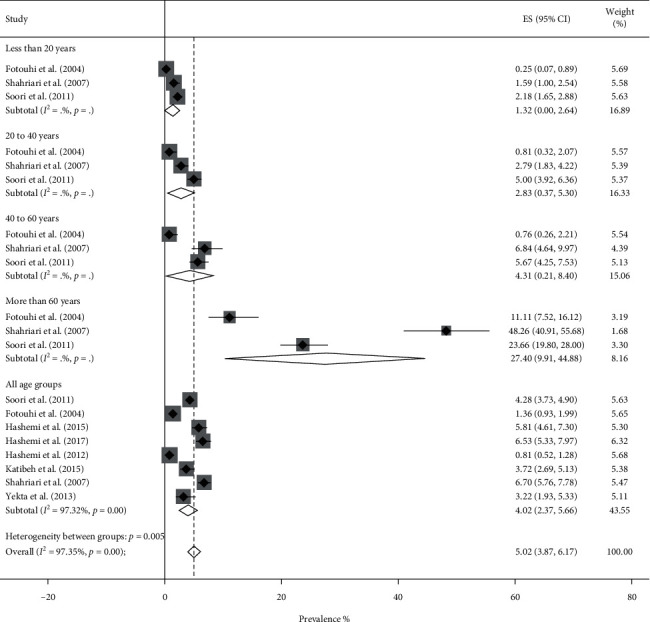
Forest plot of the prevalence of visual impairment among Iranian males.

**Figure 7:**
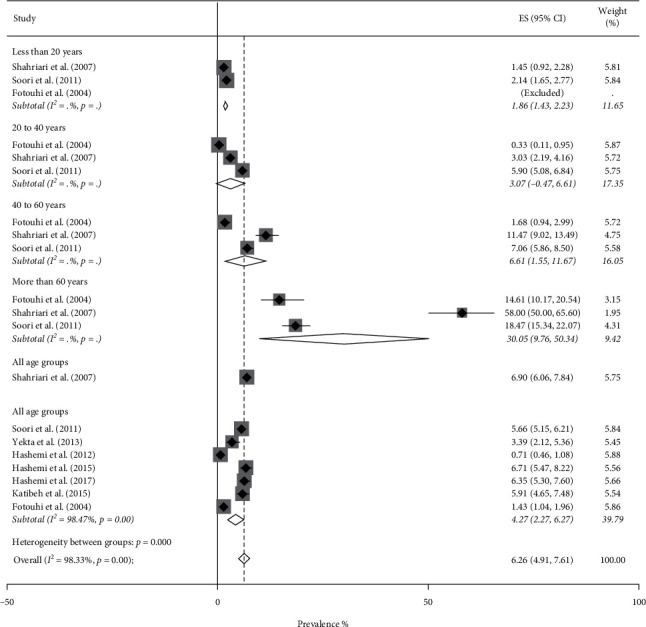
Forest plot of the prevalence of visual impairment among Iranian females.

Pooled prevalence of VI was 5.57% (95% CI: 4.71–6.43%, *Q* = 1803.98, *I*^2^ = 97.89%). It was 5.02% (95% CI: 3.87–6.17%) in men (Figure 6) and 6.26% (95% CI: 4.91–7.61%) in women (Figure 7). Based on the results obtained by the metaregression model, we found a negative independent association between higher density of pharmacists and prevalence of VI (*t* = −3.74, *P* value = 0.001).

### 3.4. VI-Related Cause

We assessed VI causes based on best-corrected visual acuity (BCVA) and presenting visual acuity (PVA), considering the applied method for measuring visual acuity in the included studies. Five studies were entered in the analysis based on BCVA and we found that the pooled prevalence of cataracts was 37.4% (95% CI: 29.5–45.3%) as the most common cause of VI. The pooled prevalence of macular degeneration was 9.7% (95% CI: 2.2–17.2%), amblyopia 8.2% (95% CI: 4.6–11.9%), corneal opacity 6.6% (95% CI: 1.9–11.2%), and glaucoma 4% (95% CI: 2.4–5.6%), based on BCVA. Other causes including vascular retinopathy (4%), lens problems (5.1%), retinal detachment (0.8%), hyperopia (3.8%), macular scar (2.4%), pterygium (0.3%), and CNS problems (7.8%) were only reported in one study; so we were not able to calculate the pooled prevalence of these causes. Moreover, the pooled prevalence of diabetic retinopathy was 17% (95% CI: 10.8–23.2%), which was reported in only two studies. The *I*^2^ was 67%, 56.3%, 79.6%, 77.8%, and 0.0% for the following causes, respectively: cataracts, amblyopia, macular degeneration, corneal opacity, and glaucoma.

Based on PVA, five studies were included in the analysis. Refractive errors were the most common causes of VI based on PVA with the pooled prevalence of 54.6% (95% CI: 43.4–65.8%). The pooled prevalence of cataract, amblyopia, macular degeneration, corneal opacity, and glaucoma based on PVA was 23.5% (95% CI: 19.1–27.9%), 4% (95% CI: 1.5–6.4%), 5.1% (95% CI: 1.7–8.4%), 1.3% (95% CI: 0.4–2.2%), and 1.8% (95% CI: 0.6–3%), respectively. The *I*^2^ in the heterogeneity test for the most common causes of VI based on PVA were as follows: refractive errors 89.4%, cataracts 48.6%, amblyopia 60.8%, macular degeneration 75.2%, corneal opacity 0.4%, and glaucoma 0.0%.

## 4. Discussion

VI and its related disabilities could be devastating, especially for children in terms of life long failure in learning, communication and employment and in the elderly population in terms of higher risk of falls and fracture [25–30]. We showed that the pooled prevalence of blindness, low vision, and VI was 0.84%, 2.7%, and 4.2%, respectively. We also found high heterogeneity among reported estimates for each of these disorders. The greater number of GPs and pharmacists was negatively associated with the prevalence of blindness and low vision, while the greater number of specialists and subspecialists was positively associated with the prevalence estimates of blindness and low vision. Specialists and subspecialists are mainly distributed in tertiary and referral centers in larger cities. In these areas, the density of persons with VI or blindness may be more than other areas.

According to the study results, higher access to healthcare (services provided by GPs and pharmacists) could result in a lower prevalence of visual impairments. It may be a result of more effective conduction of the preventive programs in the areas with a higher density of GPs. As it is clear, preventive programs and primary healthcare could prevent considerable adverse health-related outcomes.

The results of the current study indicate that the inversely significant associations were observed between the density of specialists or subspecialists and the prevalence of low vision and blindness. The findings of the study by Haghdoost et al. showed that most specialists and subspecialists work mainly in large cities and referral centers in Iran. These regions may have more densities of population suffered from blindness or low vision [14]. Notwithstanding, many other confounder variables may be present that may be ignored. We evaluated all physicians (GP, specialists, subspecialists, and pharmacists) and not only ophthalmologists in our study. Further studies should take a prospective approach to assess the association between the densities of workforces especially ophthalmologists with the prevalence of low vision, blindness, and VI in various regions.

The results of this study revealed that the prevalence estimates in our country are lower than most of the other countries and regions. A meta-analysis in six WHO regions' showed that VI prevalence was 9.2%, 9.3%, 8.2%, 9.9%, 9.8%, and 5.2% in African, Americas, Eastern Mediterranean, European, Southeast Asian, and Western Pacific regions, respectively [5]. A report from China showed pooled prevalence of blindness and low vision to be 1.7% and 4.1% [31]. In North Africa and the Middle East region, the prevalence of blindness according to age standardization was reported to be 1.1% [31]. The prevalence of blindness in our study is close to the results of the Middle East, and it is much lower than reports from African, Eastern Mediterranean, European, and South East Asia, and Western Pacific regions. Despite recent advances in preventive measures and treatment modalities following healthcare reforms in Iran, it seems that the main cause of these lower rates is the nonuniform geographic distribution of population-based studies included in this study. Another study in our region reported a pooled estimated of VI at 4.24%, which is similar to our results and a slightly higher pooled prevalence of blindness [32]. This slight difference is speculated to be due to differences in included studies as a result of various inclusion and exclusion criteria and stricter quality assessment process in our study.

We found higher VI pooled prevalence, low vision, and blindness amongst women than men. These results were in line with previous reports from other regions around the world and the other study in Iran [31–34]. A higher prevalence of blindness in female groups may be due to higher mean age and life expectancy of women relative to men's. As the age advances, the prevalence of blinding ocular diseases such as cataracts, glaucoma, and age-related macular degeneration increases. According to our study, these diseases are the main causes of blindness and VI.

Our study's result showed a higher rate of VI, low vision, and blindness in the age group older than 60 years; also age was a significant factor that causes heterogeneity in all these three outcomes. Similarly, other studies have shown that the prevalence of VI and blindness increased by increasing age [5, 34].

Since cataract is one of the main causes of VI in our study and it is considered to rise by advancing age, the high prevalence of VI and blindness by increasing age in our study can be justified by that.

Regarding the causes of VI, the most prevalent ones in our study were cataracts, macular degeneration, amblyopia, corneal opacity, and glaucoma based on BCVA, while the refractive error was the most prevalent cause of VI according to PVA. Likewise, the meta-analysis of global estimation of VI reported the proportion of refractive errors (43%) and cataracts (33%) as the main causes of VI. In addition, some other causes found in our study including macular degeneration, glaucoma, and corneal opacity were among the influential factors in this study [5]. Studies in other regions also found similar results regarding the leading causes of VI [31, 33, 35]. One study in our region that assessed the most prevalent causes of VI based only on BCVA similarly found cataracts as the main reason and reported amblyopia, corneal opacity, macular degeneration, and glaucoma among the most frequent factors resulting in VI and blindness, with a slight difference in their contribution to the proportions between the two studies [32].

According to PVA, refractive errors are the main causes of VI in our study, which can simply be corrected by using glasses and surgical correction of refractive errors, if diagnosed. Therefore, the important role of designing screening programs for detection, early diagnosis, and treatment of refractive errors in the population would be emphasized. The screening programs could be designed to be performed in schools for early detection and correction of this main cause of VI.

Prevalence of diabetic retinopathy (DR) as a cause of VI was 17%, which was reported only in two studies, while DR is one of the most important causes of VI in different parts of the world [5, 31, 33, 35, 36]. This difference could be due to lack of data from many important areas of Iran. Moreover, most studies did not report VI causes low vision and blindness separately. Also, the results of the included studies were not reported in similar age categories. Hence, desirable comparisons were somewhat difficult.

Our study had some limitations. First, there were no population-based studies from different geographical regions of Iran. Consequently, the results could not be generalized for the entire Iranian population. Further studies are needed to evaluate the main causes of blindness and its relation to the geographic distribution of some healthcare services in different geographic regions. Second, the use of different outcome measures (BCVA and PVA), and age groups in the studies made the optimal combination in the meta-analysis difficult.

In conclusion, cataracts and refractive errors were the main cause of VI in Iran, which can be corrected with simple treatment methods if healthcare services are available. Our results provide health policymakers with the opportunity to know the overall estimates of these important visual outcomes and the current discrepancies regarding visual health services in various regions of our country. However, due to the nonuniformity of data, it could be stated with caution. In consequence, further well-designed studies and national surveys should be conducted to provide accurate data from different regions of Iran.

## Figures and Tables

**Table 1:** Population-based studies reporting on the prevalence of blindness and low vision in Iranian people.

Study	Fotouhi et al. [17]	Hashemi et al. [18]	Shahriari et al. [19]	Katibeh et al. [20]	Hashemi et al. [21]	Hashemi et al. [22]	Soori et al. [23]	Yekta et al. [24]
Location	Tehran	Khaf	Sīstān va Balūchestān	Yazd	Khuzestan and Mazandaran	Shahroud	Tehran	Sari
Rural/urban status	Urban	Rural	Rural/urban	Rural/Urban	Rural	Urban	Rural/Urban	Urban
Sets, n	1	1	1	1	2	1	1	1
Study year	2002	2011	2004–2005	2010–2011	2015	2009–2010	2006	2010–2011
Population age, years	1 to +70 y/o	1 to +70 y/o	≥10 y/o	40–80 y/o	≥1 y/o	40–64 y/o	1 to +70 y/o	≥55 y/o
Sample size	6497	3475	6483	2320	3851	6311	11975	1185
Response rate (%)	70.3	45.8	84	90.4	86.5	82.2	90.4	79.1
Criteria of blindness definition	VA*∗* < 3/60 in better eye	VA < 20/400 in better eye	VA < 3/60 in better eye	VA < 3/60 in better eye	VA < 20/400 in better eye	VA < 20/400 in better eye	VA < 20/400 in better eye	VA < 20/400 in better eye
Criteria of low vision definition	3/60 < BCVA < 20/60 in better eye	VA = 20/60 or VA < 20/400 in better eye	3/60 < VA < 20/60 in better eye	3/60 ≤ BCVA < 20/60 in better eye	20/400 ≤ VA < 20/60 in better eye	20/400 ≤ VA < 20/60 in better eye	20/400 ≤ VA< 20/60 in better eye	20/400 ≤ VA < 20/60 in better eye
Visual acuity	BCVA^*∗∗*^, PVA^ǂ^	PVA	Pinhole	BCVA	PVA	BCVA, PVA	BCVA	BCVA, PVA

^*∗*^ **VA:** visual acuity, ^*∗∗*^**BCVA:** best-corrected visual acuity, and **^ǂ^ PVA:** presenting visual acuity.

## Data Availability

The data used in the analyses can be found in the published articles, which were listed in the references of this manuscript.

## Abbreviations

VI: Visual impairment
WHO: World Health Organization
IAPB: International Agency for the Prevention of Blindness
SID: Scientific Information Database
BCVA: Best-corrected visual acuity
PVA: Presenting visual acuity
GPs: General practitioners
MOOSE: Meta-Analyses and Systematic Reviews of Observational Studies
DR: Diabetic retinopathy.
